# L-Glutaminase Synthesis by Marine *Halomonas meridiana* Isolated from the Red Sea and Its Efficiency against Colorectal Cancer Cell Lines

**DOI:** 10.3390/molecules26071963

**Published:** 2021-03-31

**Authors:** Yasser S. Mostafa, Saad A. Alamri, Mohammad Y. Alfaifi, Sulaiman A. Alrumman, Serag Eldin I. Elbehairi, Tarek H. Taha, Mohamed Hashem

**Affiliations:** 1 Department of Biology, College of Science, King Khalid University, P.O. Box 9004, Abha 61413, Saudi Arabia; amri555@yahoo.com (S.A.A.); alfaifi@kku.edu.sa (M.Y.A.); salrumman@kku.edu.sa (S.A.A.); serag@kku.edu.sa (S.E.I.E.); drmhashem69@yahoo.com (M.H.); 2 Prince Sultan Bin Abdulaziz Center for Environmental and Tourism Research and Studies, King Khalid University, P.O. Box 9004, Abha 61413, Saudi Arabia; 3 Cell Culture Lab, Egyptian Organization for Biological Products and Vaccines, P.O. Box 12311, Giza, Egypt; 4 Environmental Biotechnology Department, Genetic Engineering and Biotechnology Research Institute, City of Scientific Research & Technological Applications, P.O. Box 21934, Alexandria, Egypt; tarek_micro@yahoo.com; 5 Department of Botany and Microbiology, Faculty of Science, Assiut University, P.O. Box 61413, Assiut, Egypt

**Keywords:** *Halomonas meridian*, L-glutaminase, production, 16S rRNA gene, purification, colorectal cancer

## Abstract

L-glutaminase is an important anticancer agent that is used extensively worldwide by depriving cancer cells of L-glutamine. The marine bacterium, *Halomonas meridian* was isolated from the Red Sea and selected as the more active L-glutaminase-producing bacteria. L-glutaminase fermentation was optimized at 36 h, pH 8.0, 37 °C, and 3.0% NaCl, using glucose at 1.5% and soybean meal at 2%. The purified enzyme showed a specific activity of 36.08 U/mg, and the molecular weight was found to be 57 kDa by the SDS-PAGE analysis. The enzyme was highly active at pH 8.0 and 37 °C. The kinetics’ parameters of *Km* and *Vmax* were 12.2 × 10^−6^ M and 121.95 μmol/mL/min, respectively, which reflects a higher affinity for its substrate. The anticancer efficiency of the enzyme showed significant toxic activity toward colorectal adenocarcinoma cells; LS 174 T (IC50 7.0 μg/mL) and HCT 116 (IC50 13.2 μg/mL). A higher incidence of cell death was observed with early apoptosis in HCT 116 than in LS 174 T, whereas late apoptosis was observed in LS 174 T more than in HCT 116. Also, the L-glutaminase induction nuclear fragmentation in HCT 116 was more than that in the LS 174T cells. This is the first report on *Halomonas meridiana* as an L-glutaminase producer that is used as an anti-colorectal cancer agent.

## 1. Introduction

There is an urgent need for further discovery of novel anti-tumor compounds in light of the high-risk and low potency of available drugs [1]. Colorectal cancer is the world’s third top cancer type in terms of mortality [2], while in Saudi Arabia it ranks first among males and third among females [3]. Various therapeutic strategies for colorectal cancer were used as immunotherapy, radiation, stem cells, and chemotherapies. However, an appreciable ratio of patients anguished clinically for a long period with chances of tumor recurrence with later metastasis, thus it is important to the development of new therapeutic strategies with improved clinical studies [4]. Recently, amino acid-depleting enzymes have been shown to play a prominent role in the treatment by depriving the tumor of essential nutrients. L-asparaginase was approved as an effective therapeutic against acute lymphoblastic lymphoma and auxotrophic tumors, while the therapeutic potential of arginine deiminase and L-methionase, L-arginase, lysine oxidase, L-glutaminase, and L-phenylalanine are under clinical trials [5]. L-glutaminase (EC 3.5.1.2) from various microbial sources has attracted high attention in various biological activities. The anti-tumor activity of *Alcaligenes faecalis* L-glutaminase against HeLa cell line [6], and from *Bacillus cereus* MTCC 1305 toward hepatocellular carcinoma (Hep-G2) cell line [7], were reported. L-glutaminase from *Pseudomonas* 7A has antiviral activity against retroviral disease by disruption in mRNA translation and repression of the viral replication [8]. Also, L-glutaminase from *Bacillus amyloliquefaciens* has been used as a flavor enhancer in foods [9], while the enzyme from *Bacillus cereus* LC13 showed antioxidant activity with ascorbic acid [10]. A biosensor for monitoring the L-glutamine in pharmaceutical powders was progressed by immobilizing L-glutaminase from *Hypocria jecorina* onto nanorods of zinc oxide and chitosan [11]. Glutamine is an amino acid that plays a prominent role in cellular metabolic processes, engages in ammonia formation and glycosylation reaction, and in addition, provides the nitrogen necessary for the synthesis of various nitrogenous metabolic intermediates as nucleotides, glutathione, and hexosamine [12]. The rapid proliferation of colorectal cancer cells shows more nutritional requirements. The tumor cells are auxotrophic to some nutrients such as amino acids and hence it depends mainly upon the supply of these nutrients from normal cells. The glutamine-dependent colorectal tumor cells cannot survive without exogenous glutamine [13]. The glutamine-deprivation therapy by L-glutaminase that hydrolyzes L-glutamine to L-glutamic acid and ammonia, selectively inhibits tumor growth by the blocking of de novo protein synthesis and increase of the superoxide level by oxidative stress that promotes the death of the cancer cells [7,14,15]. However, various microbial sources, such as *Escherichia coli* and *Erwinia cartovora*, were commercially able to produce L-glutaminase [12]. The chemical nature of seawater could provide microbial sources producing enzymes that could have fewer side effects when used in therapeutic applications [1]. The modified surface structure of anticancer holoenzymes due to the high salinity ensures the possibility of unique immunological properties [16]. In this regard, marine bacteria have recently attracted attention for L-glutaminase production [17]. The biological system of the Red Sea has not been investigated for its biodiversity and biotechnological importance despite it being viewed as an unprecedented marine environment for its physical and geochemical properties [18]. The exploration of distinct microbial resources with unique properties as well as the enhancement of the bioprocesses using cheap resources and kinetic parameters of purified L-glutaminase are still under development for productivity, therapeutic potential, and industrial issues [19]. The economic challenge of L-glutaminase production required the exploration of new microbial sources with high yield and large-scale production using the available cheap resources [10]. The physical and nutritional factors play a vital role in the submerged fermentation of L-glutaminase production where it depends on the presence of the appropriate substrate and feedback inhibition by the end product [20]. Accordingly, the current study focuses on the isolation and screening of L-glutaminase-producing bacteria from the Red Sea, Saudi Arabia, with an emphasis on optimizing the fermentation conditions and enzyme properties, and evaluating their effectiveness against colorectal cancer cell lines.

## 2. Results

### 2.1. Isolation and Screening of L-Glutaminase-Producing Bacteria from the Red Sea

**Figure 1:**
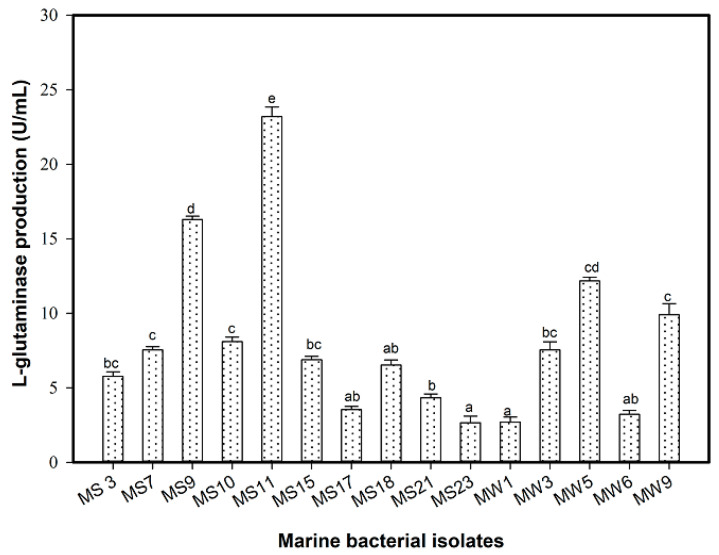
Quantitative screening of marine L-glutaminase producing bacteria. Mean ± standard error (*n* = 3) is presented. Vertical bars indicate the standard errors of the means. Means followed by different letters are significantly different at *p* < 0.05.

A total of 97 marine bacterial isolates were obtained from two marine samples; seawater and sediment collected from the Al-Marabi coast, the Red Sea, Saudi Arabia. Out of 34 L-glutaminase-producing marine bacteria, strains were detected using the rapid plate method. The number of marine bacteria able to produce L-glutaminase was higher in the sediment samples (23 isolates) than in the seawater samples (11 isolates). Of these strains, 15 isolates showed measurable pink areas around the colony (8.5–17 mm) that were proportional to their ability to produce L-glutaminase. Four unique isolates—KKU-MS11 (17 mm) and KKU-MS9 (12 mm) from sediments, and KKU-MW5 (11 mm) and KKU-MW9 (8.5 mm) from seawater samples were obtained. The quantitative screening for L-glutaminase production was evaluated using submerged fermentation that assayed spectrophotometry by using Nessler’s method. A harmonic relationship was observed between both the rapid plate method and quantitative screening, where maximum L-glutaminase produced was detected by the same four mentioned marine bacterial isolates, KKU-MS11 (23.20 U/mL), KKU-MS9 (16.30 U/mL), KKU-MW5 (12.17 U/mL), and KKU-MW9 (9.90 U/mL) (Figure 1). The high potential L-glutaminase-producing isolate, KKU-MS11 was molecularly identified and selected for further studies.

### 2.2. Molecular Identification via 16S rRNA Gene Sequencing and Phylogenetic Analysis

**Figure 2:**
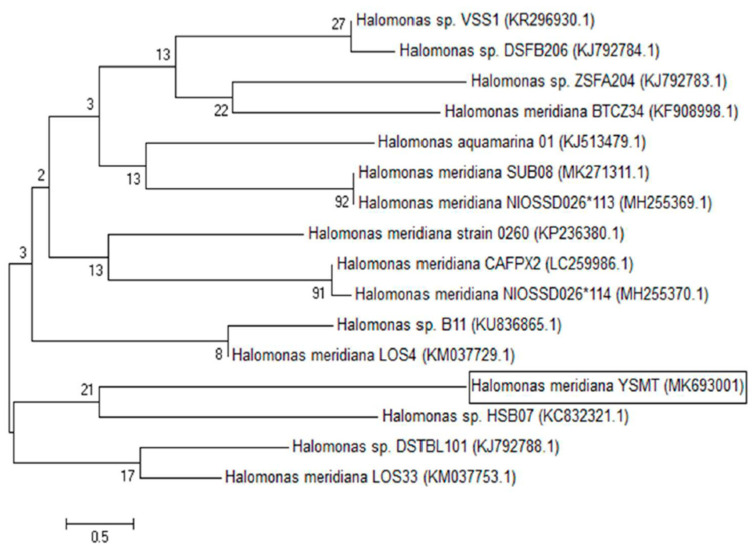
Phylogenetic tree of the *Halomonas meridian* isolate and other related strains with their accession numbers in brackets. MEGA 5.1 program was used to construct the branching pattern through the neighbor-joining tree method with 1000 bootstrap.

The PCR amplification of the 16S rRNA gene succeeded in amplifying 1500 bp of the gene using universal primers (27 F and 1492 R). The nucleotide sequences of the gene have been compared with similar sequences in GenBank and the results revealed that the isolate KKU-MS11 was similar to *Halomonas meridiana* with 100% similarity. The sequence was then submitted in GenBank as a *Halomonas meridiana* with the accession number MK693001. The phylogenetic analysis of the obtained sequence and related sequences in GenBank revealed that all the selected strains share the same ancestor followed by multiple internal nodes and branches sharing the same genus and species identification (Figure 2). 

### 2.3. Effect of Fermentation Period on L-Glutaminase Production 

**Figure 3:**
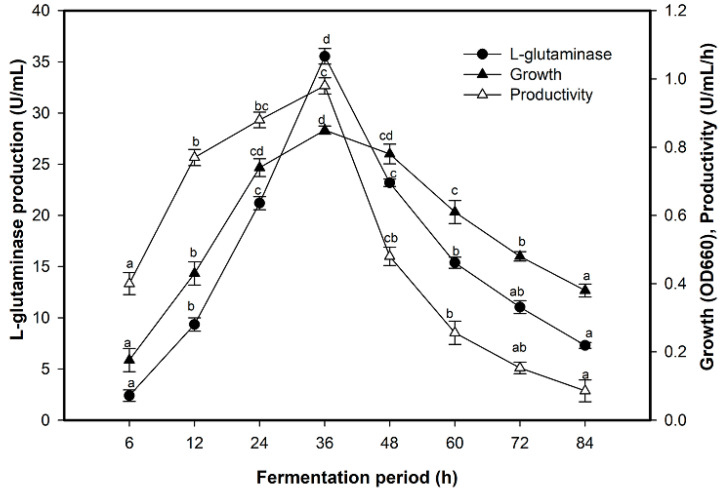
Time course of L-glutaminase production by *Halomonas meridian*. Mean ± standard error (*n* = 3) is presented. Vertical bars indicate the standard errors of the means. Means followed by different letters are significantly different at *p* < 0.05.

The time duration has a significant effect on the production of the enzyme where the bacterial growth concurrent with L-glutaminase production increased gradually over a period of up to 36 h of cultivation (Figure 3). The kinetics of bacterial growth and enzyme production demonstrated low values in their lag phase and exhibited a high level of the enzyme from 12 h at the mid-logarithm phase of growth, where at 12 h, it was 2.40 U/mL. The continuous increase in the subsequent cultures with a significant leap in enzyme production (35.54 U/mL) and enzyme productivity (0.98 U/mL/h) was observed at 36 h at the beginning of the stationary phase. An additional fermentation period beyond the optimum value decreased the enzyme production and bacterial growth rate.

### 2.4. Effect of Initial pH on L-Glutaminase Production

**Figure 4:**
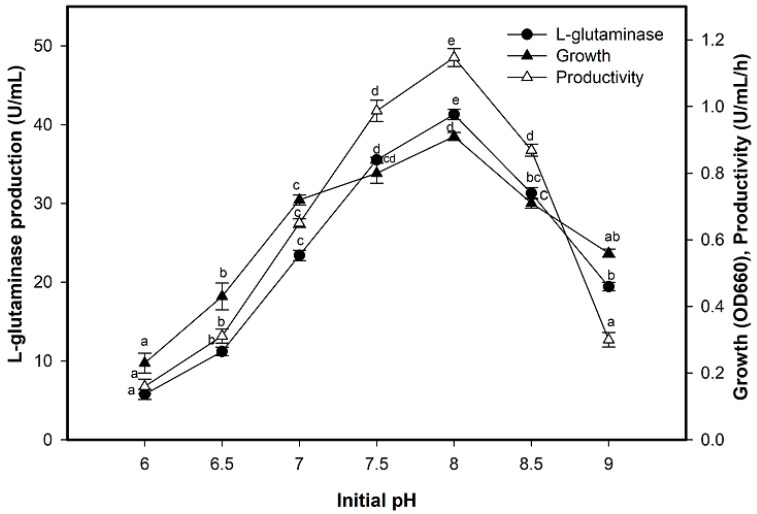
Effect of initial pH on L-glutaminase production. Mean ± standard error (*n* = 3) was presented. Vertical bars indicate the standard errors of the means. Means followed by different letters are significantly different at *p* < 0.05.

L-glutaminase production was altered according to the initial pH of the medium where it was one of the most critical parameters affecting both growth and enzyme production. The enzyme production increased gradually up to slightly alkaline conditions at pH 8.0, which was the most favorable for enzyme production (41.30 U/mL) and enzyme productivity (1.1470 U/mL/h) (Figure 4). Moreover, on the other side of pH values, the production distinctly reduced, reaching 13.94% at the acidic condition of pH 6.0, while at the alkaline condition of pH 9.0, the reduction was only 47.04%.

### 2.5. Effect of Incubation Temperature on L-Glutaminase Production

**Figure 5:**
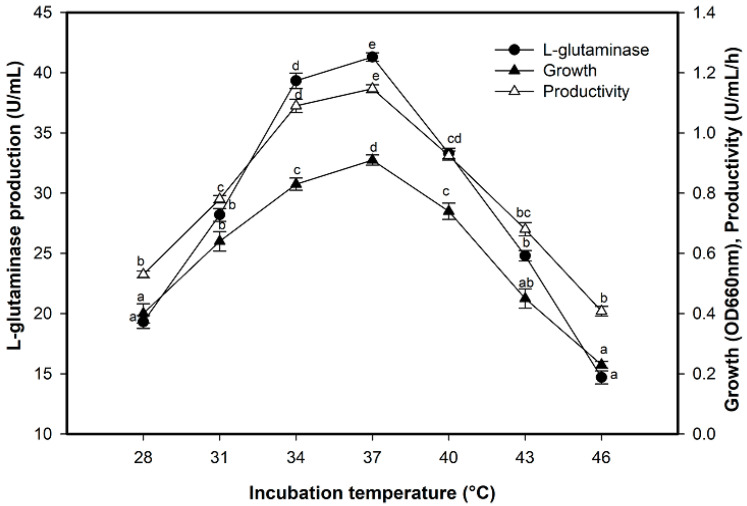
Effect of incubation temperature on L-glutaminase production. Mean ± standard error (*n* = 3) was presented. Vertical bars indicate the standard errors of the means. Means followed by different letters are significantly different at *p* < 0.05.

L-glutaminase production rates exhibited positive relation with bacterial growth as the fermentation temperature progressed (Figure 5). The indisputable correlation between enzyme production and the temperature was spotted up to 37 °C. The enzyme production was decreased when the bacterial culture was subjected to growing at temperatures other than the optimum value. The enzyme productivity was decreased by 35.57% when the incubation temperature increased from 37 °C (1.1470 U/mL/h) to 46 °C (0.408 U/mL/h).

### 2.6. Effect of NaCl on L-Glutaminase Production

**Figure 6:**
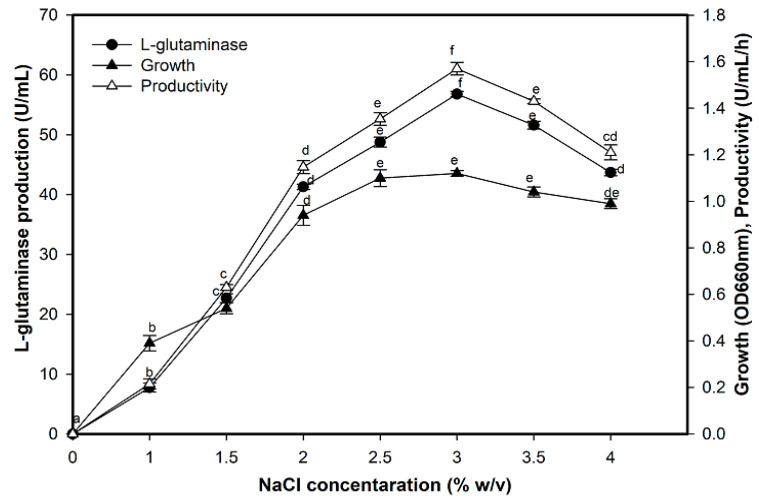
Effect of NaCl concentration (*w*/*v*) on L-glutaminase production. Mean ± standard error (*n* = 3) is presented. Vertical bars indicate the standard errors of the means. Means followed by different letters are significantly different at *p* < 0.05.

L-glutaminase productivity was influenced by salt concentration where it increased gradually when NaCl was added at various levels (Figure 6). Maximum enzyme production was observed at 3.0% where it reached 56.80 IU/mL with a productivity corresponding to 1.57 U/mL/h compared with the salt-free medium, while no significant change was observed in the growth rate at 2.5–3.5%. Besides, it was noticed that the fermentation yield was decreased beyond the optimum NaCl value reaching 43.70 U/mL and achieving a productivity of 1.21 g/L/h at 4% salt.

### 2.7. Effect of Carbon Sources on L-Glutaminase Production

**Figure 7:**
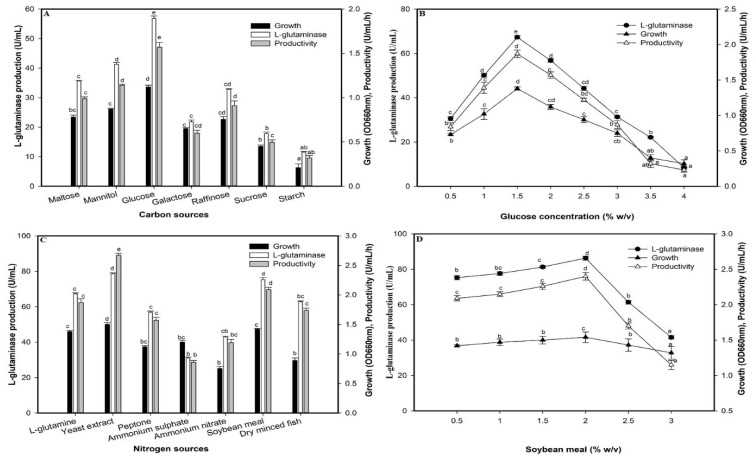
Effect of carbon sources (**A**), glucose concentration (**B**), nitrogen sources (**C**), soya bean meal concentration (**D**) on L-glutaminase production. Mean ± standard error (*n* = 3) is presented. Vertical bars indicate the standard errors of the means. Means followed by different letters are significantly different at *p* < 0.05.

The major nutritional factor playing an essential role in bacterial L-glutaminase production is a carbon source. Among the different carbon sources, glucose exhibited an enhanced effect for bacterial growth and enzyme production (56.84 U/mL), followed by mannitol (41.32 U/mL) and then maltose (35.60 U/mL), whereas sucrose and starch proved to be detrimental for the enzyme production by 68.57% and 79.69%, respectively (Figure 7A). Furthermore, optimizing the glucose concentration was carried out, where maximum enzyme production was observed at 1.5% glucose with 67.33 U/mL and productivity of 1.87 U/mL/h (Figure 7B). The additional amount of glucose in the production medium gradually showed an adverse effect on the enzyme production, which reached 8.40 U/mL at 4%.

### 2.8. Effect of Nitrogen Sources on the L-Glutaminase Production 

The efficiency of *Halomonas meridiana* in utilizing the synthetic nitrogen sources (yeast extract, peptone, ammonium sulfate, ammonium nitrate) and natural sources (soybean meal and dry minced fish) as inexpensive alternative nitrogen sources compared to L-glutamine were evaluated (Figure 7C). The yeast extract was the favored nitrogen source that enhanced L-glutaminase production by 16.84% compared to L-glutamine in the initial medium. The prominent result was that the soybean meal gave a result very close to the yeast extract (75.31 U/mL), with a decrease of only 4.98% and a rise of 11.85% over the glutamine source. Thus, soybean meal can be considered the best nitrogen source due to its availability and cheaper price. Furthermore, there was a significant decrease in enzyme productivity using the inorganic nitrogen sources; ammonium sulfate (0.86 U/mL/h) and ammonium nitrate (1.19 U/mL/h) compared to organic sources; peptone (1.57 U/mL/h) and dry minced fish (1.74 U/mL/h). Furthermore, L-glutaminase production with soybean meal as the nitrogen source was carried out with various concentrations (0.5–3.0% *w*/*v*) (Figure 7D). It was demonstrated that soybean meal significantly affected enzyme productivity with a considerable increase at an optimum concentration of 2.0%. The production of 86.33 U/mL and productivity corresponding to 2.40 U/mL/h were obtained with an increased reach of 28.21% compared to L-glutamine. The high reduction in productivity other than the optimum concentration reached 52.08% at 3% soybean meal.

### 2.9. Properties of Purified L-Glutaminase 

**Figure 8:**
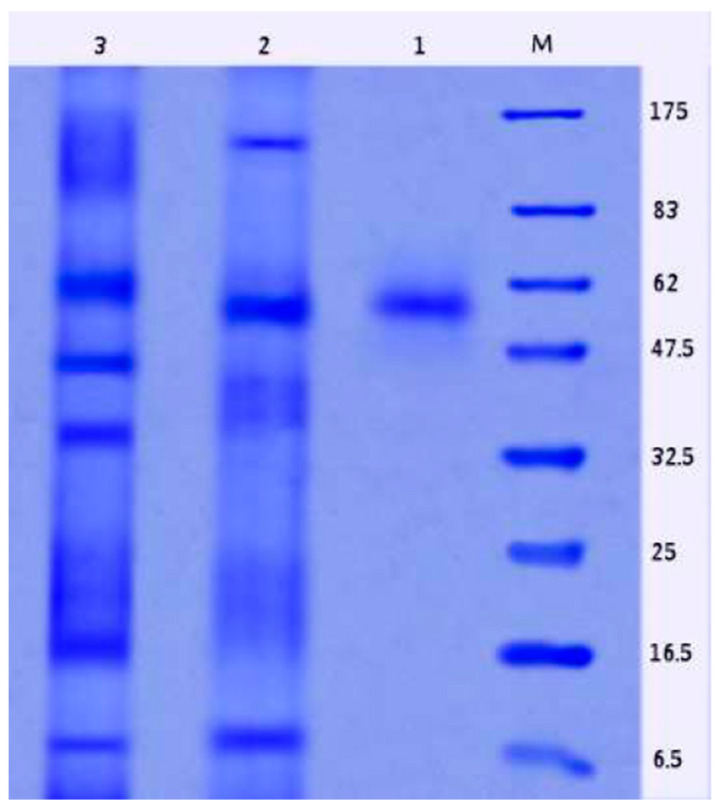
SDS-polyacrylamide gel electrophoresis of the purified enzyme. M: protein marker; 1: Sepharose 6B purified enzyme; 2: (NH_4_)_2_SO_4_-precipitated enzyme; 3: cell-free crude enzyme.

The specific activity and the purity fold of the enzyme increased with each progression of purification, although the total protein, total activity, and yield reduced relatively (Table 1). Using ammonium sulfate precipitation, the crude enzyme was purified about 2.03 times more and the yield rate was enhanced up to 78.23% with a specific activity of 22.89 U/mg. However, the yield and the final specific activity after gel filtration chromatography were 42.05% and 42.63 U/mg, respectively. The result of SDS-PAGE analysis after the final step of purification revealed a single particular protein band corresponding to the molecular mass of approximately 57.0 kDa, which exhibited the purity of the enzyme (Figure 8). 

**Figure 9:**
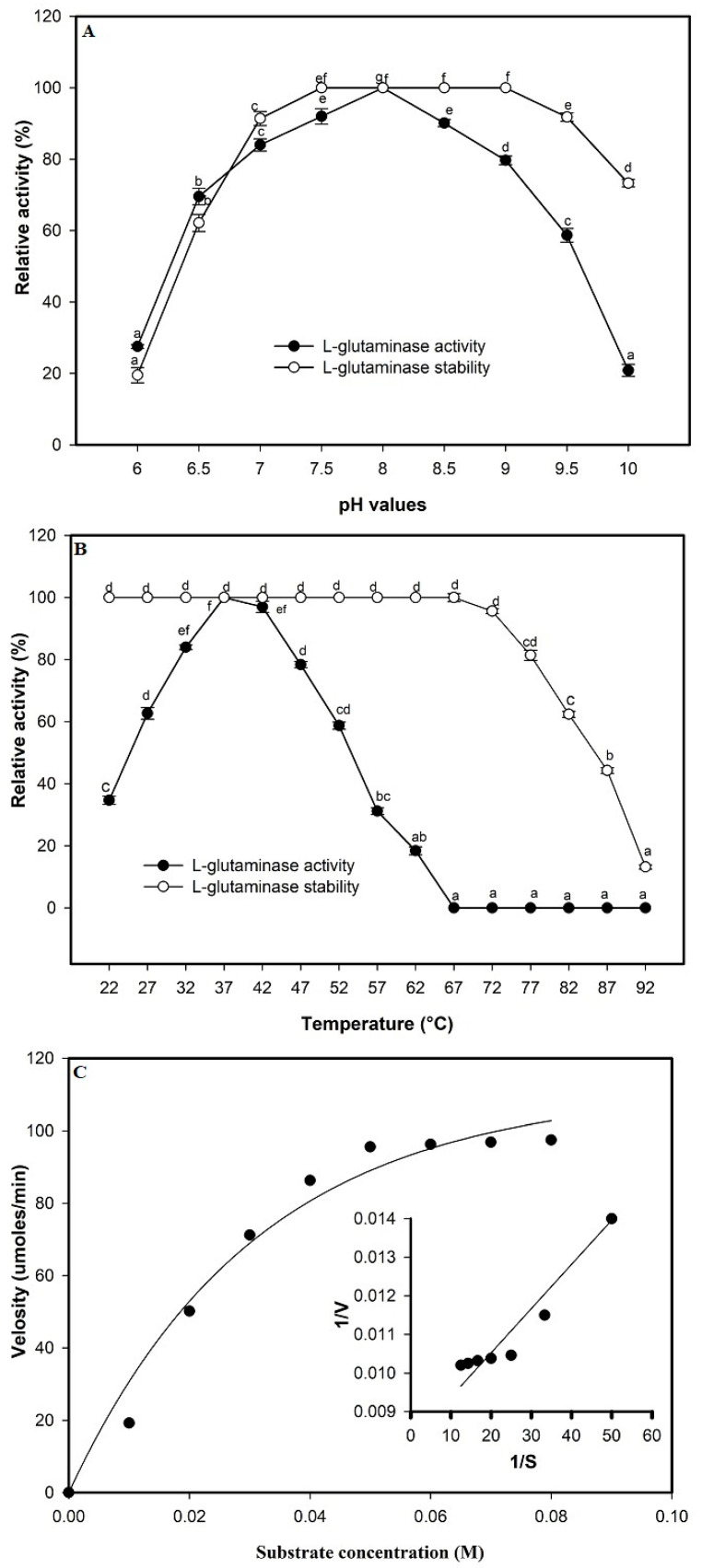
Kinetic parameters of purified L-glutaminase. Effect of pH (**A**) and temperature (**B**), on L-glutaminase activity and stability. Mean ± standard error (*n* = 3) is presented. Vertical bars indicate the standard errors of the means. Means followed by different letters are significantly different at *p* < 0.05. The reaction velocities (V) vs. substrate concentration fitted to the Michaelis-Menten equation and determination of the Km and Vmax values of the purified enzyme by Lineweaver–Burk plot (**C**).

The kinetic values of the purified enzyme were characterized by assaying the enzyme activity and stability at various pH and temperature values. The enzyme activity was observed over a pH value ranging from 7.0–8.5, and was optimum at pH 8.0; the enzyme remained stable at a pH value of 7.5–9.5. A considerable decrease was recorded in both acidic (27.50% at pH 6) and more alkaline (20.83% at pH 10) conditions (Figure 9A). Maximum enzyme activity was mentioned at 37 °C with the thermostable up to 72 °C (Figure 9B). However, a further rise in the temperature over the optimum range procures a reduction in enzyme activity. On the other hand, a gradual increase in the enzyme activity was observed with the increase in the substrate concentration from 0.01–0.07 M with optimum activity at 0.05 M. The *Km* value was calculated to be 12.2 × 10^−6^ M and *Vmax* was 121.95 μmoL/mL/min as mentioned by the Lineweaver–Burk plot (Figure 9C).

### 2.10. Cytotoxicity Assay of L-Glutaminase against Colorectal Cancer Cell Lines

**Figure 10:**
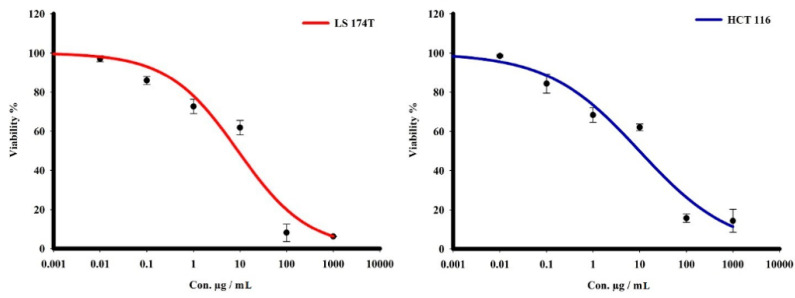
The dose-response curves of the cytotoxicity of L-glutaminase towards LS 174T and HCT 116 tumor cell lines. Cells were exposed to the enzyme with different concentrations for 72 h. Cell viability was determined by SRB stain.

The cytotoxic activities of the purified L-glutaminase were evaluated in vitro by the SRB assay towards two human colorectal cancer cells—LS 174T and HCT 116—over a concentration range of 0.001 to 1000 μg/mL. The enzyme showed a comparable cytotoxicity profile against both the tumor cells. L-glutaminase showed the most potent cell apoptosis towards colorectal adenocarcinoma cells (LS 174T) with IC_50_ 7 μg/mL. Concerning colorectal carcinoma cells (HCT 116), the enzyme showed a significantly promising cytotoxicity effect with IC_50_ 13.2 μg/mL in comparison to a positive control (doxorubicin, 200 μg/mL) (Figure 10).

### 2.11. Detection of Early and Late Apoptotic Cells

**Figure 11:**
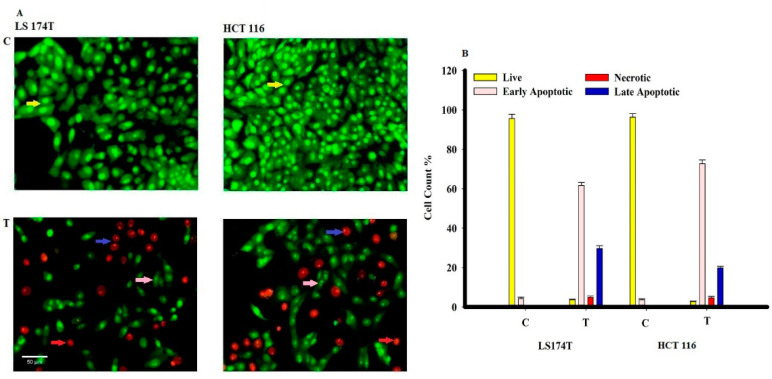
Morphological and nuclear changes (**A**), and the percentage of apoptotic cells (**B**) using acridine orange and ethidium bromide staining evaluated by the effect of L-glutaminase treatment on apoptosis of LS 174T and HCT 116 human tumor cells after 48 h treatment induced various nuclear and chromatin changes (fragmentation and condensation) at 200×. Yellow arrows indicate live cells, pink arrows indicate early apoptotic, red arrows indicate necrotic, and blue arrows indicate late apoptotic cells.

The cells after staining with AO/EtBr were categorized into four types as follows: living cells (normal green nucleus), early apoptotic (bright green nucleus with fragmented chromatin), late apoptotic (orange-stained nuclei with chromatin condensation or fragmentation), and necrotic cells (uniformly orange-stained cell nuclei). The LS 174T and HCT 116 cancer cells were labeled by an AO/EtBr dual staining 48 h extract and were treated and examined under a fluorescent microscope. Uniformly stained green with normal, round, intact nuclei and cytoplasm indicates the viability of the untreated cells (control). A high percentage of cell death was observed in early apoptotic and late apoptotic human carcinoma cells (HCT 116) more than adenocarcinoma cells (LS 174T) after treatment with L-glutaminase, while the necrotic pathway death in both cancer cells appeared in a similar percentage (Figure 11). In addition, the L-glutaminase induction nuclear fragmentation (DNA fragmented) in HCT 116 was more than the LS 174T cells. 

## 3. Discussion

The Red Sea along the coast of Saudi Arabia is proven to be a substantial source for microbial diversity with the ability to produce novel bioactive compounds. The results proved that the environment of the Red Sea was a potential source for bacterial isolates producing L-glutaminase. The ecosystem of the Red Sea, characterized by a lack of sediments and nutrient levels due to the absence of river inflow, promotes microbial diversity with unique enormous enzymatic capabilities [18]. The number of L-glutaminase-producing bacteria isolated from the Al-Marabi coast, the Red Sea, Saudi Arabia were higher in sediment samples than in the seawater samples. This is because of the dilution by the rainy season, and the pollution that affects the nutrient level [21]. L-glutaminase-producing strains were selected by a rapid plate procedure where the phenol red turns from yellow to pink under alkaline conditions as an indication of hydrolysis of glutamine to glutamic acid and ammonia [20]. The most common genetic marker for molecular identification of bacteria is the 16S rRNA gene, which is considered an accurate procedure compared to traditional methods. The most active isolate exhibiting the L-glutaminase production, KKU-MS11, was identified based on the 16S RNA gene as *Halomonas meridiana*. The potential biotechnological applications for this bacterial species were previously reported [22], while this is the first report on their ability to produce L-glutaminase. The finding of new microbial strains producing anticancer enzymes can assist with the quest for new attributes to diminish the immune reactions on account of therapy. Various marine bacterial strains producing L-glutaminase were isolated from marine habitats and identified based on 16S rRNAs such as *Bacillus subtilis* [23], *Bacillus cereus* MTCC 1305 [14], *Aeromonas veronii* [24], *Providencia* sp. [25], *Acinetobacter calcoaceticus* PJB1 [19], and *Halomonas* [16]. The enzyme productivity of L-glutaminase by *Halomonas meridiana* increased 2.68-fold after the optimization of the fermentation process. The enzyme production was observed to be dependent on microbial growth where the isolate was capable of producing L-glutaminase after 36 h of fermentation in the late logarithmic growth phase. The short fermentation period for enzyme production by *Halomonas meridian* as compared with other microbes exhibited potential productivity in an inexpensive process. The optimum fermentation period for L-glutaminase production varied depending on the bacterial strains, and most of them were at the beginning of the stationary phase of growth. The maximum L-glutaminase production by *Brevundimonas diminuta* was after 28 h [26], at 48 h of incubation by *Streptomyces griseus* [27], and at 40 h by *Bacillus cereus* MTCC 1305 [28]. The extension of the fermentation period reduces the enzyme production due to the productivity associated with bacterial growth and the catabolite repression by the final product, glutamate [29]. The initial pH of the fermentation medium plays the main role in the bioavailability of trace minerals and the metabolic synthesis of enzymes [10]. Considering this, maximum enzyme production was accomplished at pH 8.0, which was in concert with the pH recorded in the marine samples (pH 8.2). The bacterial growth and enzyme production were optimal at pH values ranging from 7.0 to 8.5, which can be elucidated by bacterial adaptation to unpropitious conditions [30]. On the other hand, the effect of fermentation temperature on bacterial growth and enzyme production could be ascribed to the effects on the enzymes involved in L-glutaminase synthesis [26]. Optimum enzyme production and growth were obtained at 37 °C, and the enzyme production decreased when the microbial culture was subjected to growing at temperatures higher than the optimum value, referring to the mesophilic nature of the strain. The results were in congruence with *Bacillus subtilis* RSP-GLU at 37 °C and pH 7.24 [29]. Our results were highly comparable with Singh et al.’s study on *Vibrio costicola* [31], which produced L-glutaminase optimally at pH 7.0 and 35–37 °C, *Streptomyces* sp. at pH 7.0 and 30 °C [32], and *Bacillus cereus* MTCC 1305 at pH 7.5 and 34 °C [28]. The growth of the marine bacterium, *Halomonas meridiana*, was not observed at 0% NaCl, which confirms that the strain was related to obligate halophiles. In this concern, many reports mentioned that *Halomonas meridiana* is a slight halophile with optimal growth at 1.0%–3.0% NaCl and a halotolerant range of 0.5%–22.5% [33]. Maximum enzyme production was observed at 3.0% (56.80 IU/mL) and no significant change in the growth rate was observed at 2.5%–3.5%, which corresponded with the salinity measured in the marine samples (33.1 ppt). The results were compared with the enzyme obtained at 4% NaCl by *Streptomyces griseus* (45 IU/mL) [27] and at 0.1% NaCl by Streptomyces sp. (32.5 U/mL) [30]. The highest L-glutaminase production was achieved using glucose as a carbon source at 1.5%. While glucose promotes enzyme production in *Vibrio costicola* [31], *Providencia* sp. [34], and *Beauveria* sp. [35], it repressed the enzyme production by *Stenotrophomonas maltophilia* NYW-81 [36]. The results show that mannitol was almost as good as glucose, but sucrose and starch were not preferred for L-glutaminase production. In other studies, galactose was the optimal carbon source for *Streptomyces griseus* [25], fructose for *Bacillus subtilis* JK-79 [30], rhamnose for *Bacillus subtilis* OHEM11 [37], and maltose for *Bacillus cereus* [38]. The nitrogen source is an important nutritional factor that affects growth and enzyme production. The yeast extract was optimum compared to other synthetic and natural nitrogen sources. Although some research mentioned that the yeast extract enhanced the L-glutaminase production by *Zygosaccharomyces rouxii* [34]; others reported that malt extract was optimal for *Streptomyces rimosus* [39] and L-glutamine for *Bacillus cereus* LC13 [36]. Despite the soybean meal having a result very close to the yeast extract with a decrease of only 4.98% in enzyme production, it caused an increase in enzyme production when used at 2% and reached 86.33 U/mL, equivalent to 9.73%. Thus, soybean meal can be considered as the best nitrogen source due to its availability and cheaper price. L-glutaminase production by *Streptomyces griseus* was optimal while using 1.0% glutamine plus 1.0% yeast extract, where the yeast extract served as a complex nitrogen source for metabolic activity and glutamine stimulated enzyme production [27]. In our study, soybean meal may act as a complex nitrogen source and stimulator for enzyme production as it contains about 18.82% glutamine [40]. In this context, various studies have focused on the production of bio-products from soybean meal [41], while no reports were mentioned for its potential uses for L-glutaminase production. The enzyme purity was increased gradually by ammonium sulfate precipitation, dialysis, and sephadex-200 [42], and it reached a yield and specific activity of 42.05% and 42.63 U/mg, respectively, in accordance with other reports [32,43]. The molecular weight of *Halomonas meridiana* L-glutaminase (57.0 kDa) estimated by the SDS-PAGE analysis varied depending on the microbial enzyme source—*Bacillus subtilis* OHEM11 (54.8 kDa) [37], *Bacillus cereus* LC13 (35.0 kDa) [38], and *Streptomyces canarius* FR (44.0 kDa) [43]. The optimum temperature and pH for L-glutaminase activity was comparable to the physiological situation of the human body [16], where the high activity and stability occurred at pH values of 8.0 and 9.0, respectively, with maximum activity at 37 °C and complete thermal stability at about 72 °C for 1 h. The alkaline character of the L-glutaminase confirms its carcinostatic behavior because it is considered the main physiological effect for anti-tumor activity [37]. The results were comparable with L-glutaminase produced by the optimal activity of *Bacillus cereus* LC13 at pH 7.0 and 37 °C [38], and the maximal activity of *Streptomyces canarius* FR L-glutaminase at pH 8.0 at 40 °C and its stability at a wide range of pH from 5.0–11.0 and thermal stability up to 60 °C [43]. The purified L-glutaminase has a lower *Km* value of 12.2 × 10^−6^ and a *Vmax* value of 121.95 μmol/mL/min, indicating a high affinity to the substrate. The results can be compared with the kinetics parameters of L-glutaminase from *Streptomyces* sp. [32] and *Bacillus cereus* LC13 [38]. Colorectal cancer is the third major worldwide cancer after lung and breast cancer, so the potential chemotherapies for its treatment are under development and clinical trials constantly [2]. Subsequently, there is an imperative necessity to discover new drug sources with more efficient therapeutic properties. The unique properties of the Red Sea environment promote a unique chance to yield bacterial L-glutaminase with unique therapeutic properties [1,26]. The current study demonstrated the high marine *Halomonas meridiana* L-glutaminase effect in colorectal cancer cell lines which are linked with the repression progression of tumor cells. Two types of human colorectal cancer cells were tested—Dukes’ type B, colorectal adenocarcinoma (LS 174T), the cell line used for the expression of diverse oncogenes, and colorectal carcinoma, HCT 116, used for colon tumorigenicity studies [44]. L-glutaminase showed the most potent cell death effect towards colorectal adenocarcinoma cells, LS 174T (IC_50_ 7.0 μg/mL,) than HCT 116 (IC_50_ 13.2 μg/mL). L-glutaminase plays a crucial role in catalyzing glutaminolysis, and its expression is often increased in tumors [45]. It is known that the initiation and proliferation of tumors are associated with the changes in the metabolism of cancer cells that exhibit more reliance on glutamine for proliferation and development [46]. Our results were in agreement with the anticancer efficiency of *Streptomyces canarius* L-glutaminase, which was highly active against HepG2 (IC50, 6.8 g/mL) and HeLa (IC50, 8.3 g/mL) cell lines and had no effect on MCF7 cells, while the HCT-116 (IC50, 64.7 g/mL) and RAW264.7 (IC50, 59.3 g/mL) cells showed moderate cytotoxic effects [43]. Also, the cytotoxic activity of *Staphylococcus aureus* L-glutaminase against the colorectal adenocarcinoma (LS 174T) cell line was inhibited with an IC_50_ of 37.19 IU/mL [13]. The cytotoxicity effect of L-glutaminase exhibited a respectable anticancer activity against the NFS-60 (IC_50_ value 6.95 g/mL), HepG2 (IC_50_ value 17.67 g/mL), and MCF-7 (IC_50_ value 10.89 g/mL) cancer cell lines [37]. Also, the influence of enzymes in colorectal cancer was previously studied by evaluating the proliferation and cell death in colorectal cancer cell lines [6]. The viability test is the relationship of cells with drug toxic effects where the reduction in MCF-7 cell line viability, increased in a dose-dependent pattern by increasing the concentration of L-glutaminase [23]. The decrease in the cell line viability as the effect of enzyme treatment could be ascribed to the downregulation of the telomerase activity of the tumor cells [47]. Whereas a higher rate of cell death was observed with early apoptosis in HCT 116 compared to LS 174 T, while late apoptosis was observed in LS 174 T more than HCT 116 after treatment with L-glutaminase, the necrosis pathway death in both cancer cells appeared in a similar proportion in the same trend as Elbehairi et al. [48]. L-glutaminase converts the amino acid L-glutamine into L-glutamate and ammonia. It selectively targets cancer cells by depleting the amino acids that the cancer cell is unable to prepare [12]. Finally, this is the first report on L-glutaminase production from the marine bacterium *Halomonas meridiana* and its anti-colorectal cancer efficiency. Nevertheless, further studies are required on the enzyme in vivo therapeutic efficiencies such as the kinetic parameters, reduction of antigenicity, half-life determination assays, pharmacokinetic, and pharmacodynamics profiling in animals, and human clinical trials for enhancing their medical applications.

## 4. Materials and Methods

### 4.1. Marine Sample Collection

The seawater and sediment samples were collected between January and March 2019 from the Al-Marabi coast, the Red Sea, Saudi Arabia (16°47′39″ N 42°47′25″ E). The samples were kept at 4 °C until processed. The physical parameters of the samples, pH (8.1), temperature (36 °C), and salinity (33.1 ppt) were estimated using Portable Meters (OAKTON, USA). 

### 4.2. Isolation and Selection of L-Glutaminase Producing Bacteria 

The marine samples were serially diluted, and 0.1 mL of the dilution was spread on the Modified M-9 agar medium containing (g/L): KH_2_PO_4_, 3.0; L-glutamine, 5.0; NaCl, 20.0; MgSO_4_.7H_2_O, 0.5; CaCl_2_.2H_2_O, 0.15; Na_2_HPO_4_.2H_2_O, 6.0; glucose, 20.0 g and agar, 15.0 g, and supplemented with 0.012 g phenol red as the pH indicator (pH 6.8) [17]. The rapid plate screening method was performed for 48 h of incubation at 37 °C, and the change of color around the colonies from yellow to pink was translated as a positive response to the L-glutaminase production. The quantitative screening was carried out in a shaking incubator (Shell Lab, SSI5, Cornelius, NC, USA). The 5 mL inoculum (1 × 10^8^ cells/mL) of the 24 h prepared culture was incorporated into 250 mL Erlenmeyer flasks at 37 °C for 72 h at 150 rpm. The culture was centrifuged at 8000 rpm for 15 min and the crude enzyme activity was measured at 540 nm. 

### 4.3. Molecular Identification of the Promising Bacterial Isolate 

The genomic DNA extraction of a high L-glutaminase-producing isolate was achieved using the QIAamp DNA Mini kit (Qiagen Inc., Valencia, CA, USA) [49]. The amplification and sequencing of the 16S rRNA gene were accomplished using universal primers—27 F (5′ CCA GCA GCC GCG GTA ATA CG 3′) and 1492 R (5′ ATC GG(C/T) TAC CTT GTT ACG ACT TC 3′)—using the PCR Master Mix kit (TAKARA, Japan). The amplified gene was subsequently sequenced (Macrogen, Korea), and the obtained sequence was submitted in GenBank (http://blast.ncbi.nlm.nih.gov/Blast.cgi) using the new accession number. Moreover, the sequence was matched with other related sequences in GenBank and their alignment and phylogenetic relationship were constructed using the MEGA 5.1 program.

### 4.4. Optimization of L-Glutaminase Production

The enzyme production was carried out in 250 mL Erlenmeyer flasks containing 50 mL of Modified M-9 broth medium at 37 °C at 250 rpm. The cell suspension of 24 h grown in 50 mL of the medium was used as the inoculum (10% *v*/*v*). Thereafter, the centrifuged fermentation broth (8000 rpm/20 min) was utilized as the crude enzyme and the bacterial growth was monitored as an OD (660 nm). The optimization of fermentation parameters of enzyme production was conducted at different incubation periods, pH values, temperatures, carbon sources, and nitrogen sources by a one factor at a time approach. 

### 4.5. L-Glutaminase Purification and Its Characterization

The ammonium sulfate (70%) was added to the crude enzyme and incubated overnight at 4 °C. The precipitated mixture after centrifugation (4000× *g* for 20 min) was dialyzed against the Tris-HCl buffer (50 mM) overnight at 4 °C and subjected to a Sephadex G-200 column. The bound protein fractions were assayed for L-glutaminase activity and the main fraction was lyophilized. Sodium dodecyl sulfate-polyacrylamide gel electrophoresis (SDS-PAGE) was performed and the molecular weight of L-glutaminase was determined using standard molecular weight markers (Bio-Rad, Hercules, CA) [37]. The purified enzyme was characterized for its activity and the stability of temperature (22–92 °C) and pH (6.0–10.0). The effect of L-glutamine concentration at 0.01 M–0.8 M on enzyme activity was assayed and the kinetics parameters of *Km* and *Vmax* of the enzyme were estimated by the Lineaweaver-Burk plots. 

### 4.6. Analytical Methods

The L-glutaminase activity assay was measured using the nesslerization method [31]. A total of 50 μL of the enzyme was mixed with 450 μL of Tris-base (50 mM, pH 7), and 0.5 mL of glutamine (0.04 M). After 30 min of incubation at 37 °C, the mixture reaction was stopped by the addition of 0.5 mL of 15% (*w*/*v*) trichloroacetic acid. After centrifugation (8000 rpm, 15 min), 1 mL supernatant was mixed with 1.0 mL distilled water and 250 μL Nessler’s reagent and incubated for 10 min. The enzyme activity was recorded using a spectrophotometer at 490 nm. The unit of the enzyme was defined as the amount of enzyme that released 1 µmol of ammonia per minute. The protein concentration using bovine serum albumin as the standard was measured according to the Lowry method [50]. 

### 4.7. Anti-Colorectal Cancer Assay of L-Glutaminase

#### 4.7.1. Cell Culture

Colorectal adenocarcinoma, Dukes’ type B (LS 174T), and colorectal carcinoma (HCT 116) human cell lines were obtained from the American Type Culture Collection (ATCC). Cells were maintained in RPMI-1640 supplemented with (100 μg/mL) penicillin (100 units/mL) and heat-inactivated fetal bovine serum (10% *v*/*v*) in a humidified, 37 °C, and 5% (*v*/*v*) CO_2_ atmosphere [51].

#### 4.7.2. Cytotoxicity Assay of L-Glutaminase

The cytotoxicity of the L-glutaminase produced from *Halomonas meridiana* was evaluated against (LS 174T and HCT 116) human tumor cells using Sulphorhodamine B assay (SRB). Eighty percent of the confluence growing cells were trypsinized and cultured in a 96-well tissue culture plate for 24 h before treatment with the enzyme. The cells were treated with various concentrations of enzymes (0.01–1000 µg/mL) with a control of untreated cells. The cells were incubated with the enzyme for 72 h and subsequently fixed with TCA (10% *w*/*v*) for 1 h at 4 °C. After several washing cycles, the cells were stained by a 0.4% (*w*/*v*) SRB solution for 10 min in a dark place. After drying overnight, the SRB-stained cells were dissolved with Tris-HCl and the color intensity was measured in a microplate reader at 540 nm. The viability percentage of each tumor cell line in relation to enzyme concentrations was analyzed to get the IC_50_ value using the Sigma Plot 12.0 software [52].

#### 4.7.3. Detection of Early and Late Apoptotic Cells

The DNA binding dyes, acridine orange and ethidium bromide (EtBr) were used for the morphological detection of live, apoptotic, and necrotic cells. AO is taken up by both non-viable and viable cells that emit green fluorescence when intercalated into DNA. EtBr is taken up only by non-viable cells whereas it is excluded by viable cells and emits red fluorescence by intercalation into DNA. The cells were seeded on a cover slide inside a six-well plate. The cells were incubated in a CO_2_ incubator at 37 °C temperature and 5% CO_2_ for 24 h, and then treated with pre-computed IC_50_ concentrations of enzyme and incubated for 48 h. The cells were stained with a mixture of acridine orange 100 μg/mL/ethidium bromide (AO/EB) 100 μg/mL in PBS 1x on each well and then incubated for 5 min in RT. The cover slides were placed with cultured stained cells on slides, which were examined using a fluorescence microscope [53]. 

### 4.8. Statistical Analysis

The one-way ANOVA was investigated and the significant differences at *p* < 0.05 were assayed utilizing Minitab (version 15). The standard error of the mean for *n* = 3 was represented by the error bars. Means followed by different letters were significantly different at *p* < 0.05. 

## Figures and Tables

**Table 1:** Purification procedure of crude L-glutaminase from *Halomonas meridiana.*

Purification Procedure	Enzyme Activity (U)	Protein (mg)	Specific Activity (U/mg)	Fold Purification	Yield (%)
Cell-Free Extract	8633	764	11.29	1.0	100
Ammonium Sulphate	6754	295	22.89	2.03	78.23
Sephadex G-200 Column	3631	85.17	42.63	3.77	42.05

## Data Availability

All data are available upon request from the authors.

## Sample Availability

Samples of the compounds are available from the authors.
